# Extra-Limites

**Published:** 1833-10-01

**Authors:** 


### EXTRA-LIMITES.

*To the Editor of the Medico-Chirurgical Review.**

The last number of this Journal contains a review of a work recently published by me, entitled "A Treatise on the Urethra; its Diseases, especially Stricture, and their Cure." Had the reviewer contented himself with criticising the opinions or disputing the positions I advanced, I should not have been induced, either by crude observations, fallacious reasoning, or ill-natured comment, to intrude myself upon your attention. He

---

* Mr. Phillips complains that he is injured by the review of his book on Stricture, and as the Editor never wilfully injured an author, he has readily given insertion to to Mr. Phillips' reply. The charge of ignorance or wilful misrepresentation made against the reviewer, must be met by the reviewer himself, and there the matter must end.—Ed.

has however thought fit to charge me with a moral offence. I am accused of a literary fraud. I deny the imputation, and I ask of you the justice to make my denial as public as the accusation.

The charge of the reviewer amounts to this, that I have endeavoured to palm upon the public as an original invention of my own, an apparatus for cauterizing the urethra, which, to use his own language, is not merely an imitation of M. Ducamp's, but is actually the thing itself.

Basing himself upon this assumption, the reviewer has directed by turns, his wit and his indignation against the dishonest plagiarist; but I hope to satisfy your readers, that his wit has been misplaced; his indignation premature; and that, in hazarding an erroneous and groundless imputation, he has exposed himself to grave censure. In complaining of the injustice that has been done me, I desire not to impute unworthy motives to another, and least of all to a man of whom I know nothing, and to whom I am perhaps unknown.

It is however certain that the reviewer either did not understand the subject, and was therefore incompetent for the task he undertook to perform, or wilfully misrepresented what he really understood. This dilemma has not been produced by me; it has been occasioned by the reviewer himself, and he may take which alternative he pleases.

After the unqualified accusation in which he has indulged, the reader will be surprised to find that in no one page of my treatise have I arrogated to myself the merit of inventing, improving, or modifying the caustic apparatus introduced by Ducamp.

In treating on cauterization of the urethra, I stated that the objections to the old modes of applying caustic were, the difficulty of confining its action to the diseased portion of the canal—the escape of the caustic from the instrument, or its dissolution by the urethral mucus—and the necessity, from the imperfection of the instruments employed, of making the application to the anterior portion of the stricture.

I then added, p. 206, and 207, "The method of cauterization introduced by Ducamp and modified by Lallemand undoubtedly limits the application of the caustic to the particular point we wish, and presents, so far, all the advantages of which the operation would seem susceptible.

The operation is performed by introducing the caustic, protected by a canula, within the stricture, and cauterizing from within outwards."

This passage might surely be considered no very ambiguous recognition of the claims of Ducamp—no niggardly admission of a merit, it never entered into my imagination to contest: but as further evidence of my feelings, I may perhaps be allowed to cite the following extract from the preface to my work; where, after expressing my obligations to Ducamp amongst many other writers, I add the following words—"To any other writer from whom I may have derived assistance of which I am at this moment unconscious, I beg likewise to make my sincere acknowledgments, feeling as I do, that there is no species of property which should be held more sacred than the conceptions of the mind."

Surely this is a feeling little compatible with wilful and acknowledged plagiarism.

In p. 217, I recommend the use of a cutting instrument to assist the passage of the caustic apparatus in cases where the caustic cannot otherwise be introduced, and in p. 221—2, I recommend the introduction in certain cases of a cutting instrument by which the stricture may be removed without the aid of caustic.

Now those are the only urethral instruments of which I have claimed the invention, and I have yet to learn (what indeed is not asserted by the reviewer) that I am not entitled to the merit (if any) which may attach to that invention.

The reviewer has copied my description of the model sound and contrasted it with Ducamp's description of the exploring catheter, and upon the resemblance of those instruments seems to have mainly rested his charge of plagiarism.

Where however does he find any claim put forward by me to the invention of the sound? Does the introduction of a drawing of the instrument in a diagram at the end of the work constitute an appropriation by me of the invention? It might as well be said that the author of a work on surgery claims to be the inventor of all the instruments of which his work may contain a description.

Ducamp may with as much justice be charged with plagiarism, for the model sound or exploring catheter, by whichever name it may be called, is, with slight modifications, the copy of an instrument used in the 16th century by Germain and Mazelt, and later by Lemonier.

The reviewer says, the caustic apparatus is a copy of that of M. Ducamp, "but the length of M. Ducamp's description and the brevity of Mr. Phillips' prevent us from placing side by side this 'counterfeit presentment of two brothers.'"

Can it be possible that the author of this unworthy insinuation had seen the express declaration by me. that we owe the improved caustic apparatus to Ducamp and Lallemand. If he had, then indeed reviewing may truly be termed the ungentle craft.

I never once intimated that the caustic apparatus recommended by me possessed any advantage over that of Ducamp. I feel no wish to direct attention to the subject. I am now however compelled to speak out, and will shew the reviewer, that the caustic apparatus introduced by me is not a counterfeit presentment of that of Ducamp. To do this I must describe both instruments, and I shall begin with that of Ducamp, which he called a porte-caustique.

The porte-caustique is composed 1st. of an elastic gum tube of the size of a No. 7 or 8 bougie, one end of which is tipped by a rim of platina of the same diameter with the tube and of the length of about six lines—this constituted the beak of the instrument. The centre of the beak presents an orifice of rather more than a line in diameter.

2ndly. Of a fine elastic gum bougie longer than the tube into which it is introduced and terminated at one extremity by a cylinder of platinum five lines in length, and at least a line in diameter, presenting a cauliculated furrow three lines long, in which the caustic is impacted by fusion.

This cylinder fills up the orifice at the beak of the tube, which is introduced into the urethra, and when in contact with the stricture the bougie is projected beyond the beak of the tube into the orifice of the stricture—a certain number of revolutions are performed with the bougie, for the purpose of applying the caustic to the indurated surface; the bougie is then returned into the tube and the instrument withdrawn.

Such is the apparatus of Ducamp. This apparatus, ingenious as it certainly is, left much to be desired.

Thus when the stricture was long the treatment was protracted, because the porte-caustique acted only to an extent of four lines—and the bougie could not be advanced until the strictured portion of the canal was sufficiently large to permit the canula to pass. A long time too occurred occasionally before the patient found any relief; which he could not do until the caustic had been applied upon the most profound part of the stricture.

However frequently the application may have been made, the urethra could not be restored to its original diameter, for as soon as the canal was sufficiently enlarged to permit the passage of the tube, the parietes of the urethra were removed by it so far apart that they could not be acted upon by the caustic.

The flexibility of the instrument and especially of the bougie which supports the caustic, produced too, occasional inconveniences, for although the resistance occasioned by the stricture might be inconsiderable, the bougie was sometimes bent back in the canula —the cauterising portion was not projected beyond the tube, and cauterisation was not effected—or, if, as occasionally happened, the bougie did not enter the orifice of the stricture—the caustic was deposited on the healthy portion of the canal anterior to the induration, whilst no sensation was communicated to the hand of the operator to apprise him he was going wrong. The caustic too was not sufficiently protected from the mucus or blood that might be met with in the urethra, and by which it was sometimes dissolved before it could be brought into contact with the stricture.

These then are the prominent defects of the porte-caustique, and these I endeavoured to correct by the caustic apparatus I recommended, and which I shall now describe.

It consists 1st. of a platina canula, either straight or curved; its volume varying from No. 1 to No. 9 or 10, its diameter from one to three lines. This canula is open and of the same diameter at each extremity, perfectly cylindrical and intended to protect the caustic. 2ndly. Of a platina tube, longer than the canula, terminated at one end by a slight bulb formed so as exactly to block up one of the extremities, but not to project beyond the circumference of the canula, and protecting the caustic from the mucus and blood contained in the urethra.

The portion of the tube of which this bulb is a part, is from ten to twenty lines in length, and is screwed on the remaining portion; it presents a canal varying in length from 6 to 16 lines, in which the caustic is impacted by fusion. The flexibility of Ducamp's apparatus is corrected by the metallic character of mine; the confined range of his instrument is exchanged for an extended action,—the caustic is brought with more certainty and safety into contact with the stricture, and is always protected from the mucus and blood found in the urethra.

By means of this instrument we may explore the canal—cauterize the stricture at once in all its extent—or passing through a stricture of inconsiderable character, go more profoundly and attack another more considerable; and thus treat two or more strictures at the same time, affording the patient prompt relief, and cauterizing only the diseased portion of the canal.

Ducamp's *principle* of applying caustic was similar to that recommended by me—his *mode* of doing so possessed the defects I have pointed out.

He thought that, after the orifice of the stricture had been enlarged to a certain extent by cauterization, the cure should be completed by dilatation.

I maintain that total destruction of the induration is absolutely required before a cure can be effected, and that consecutive dilatation is unnecessary, and cannot complete the cure.

Are then the principles of treatment similar?—Are the modes of application similar? —Are the porte-caustique of Ducamp, and the caustic apparatus recommended by me "a counterfeit presentment of two brothers"?

Even if all these questions could be answered in the affirmative, the term plagiarist affixed to me by the reviewer would yet be inapplicable.

I claimed no invention that does not belong to me:—I claimed neither the invention, nor the improvement of the caustic apparatus. I ascribed the invention to Ducamp, and the modification to Lallemand, but I may now ask whether I have not effected at least some improvement of that instrument.

I have not appropriated to myself the model sound, neither do I, with the reviewer, ascribe its invention to Ducamp.

When he next treats of the sound he will know that it belongs to other men and other times, and that it has been known to the profession for more than two centuries.

I commenced this article by denying the truth of the imputation of the reviewer:—I have now I trust proved the correctness of that denial; and shall willingly abide the decision of the profession on the question at issue between us.

It is somewhat singular, that whilst accused in an English periodical of injustice to the claims of a Frenchman, my work should have been reviewed in a French periodical of eminence in terms highly flattering to the author; and should be strongly recommended to the attention of the medical profession of that country. When I sat down to the composition of that work, I sought to do no more than to place before the profession the opposite opinions of the greatest men in our own and other countries, on the treatment of a distressing class of diseases, regarding which much contrariety of opinion existed—to balance fairly and dispassionately the merits of rival systems—and to state the objections, as they appeared to me, to each.

I pretended to no originality of conception, to no novelty of idea—part of my treatise was historical, part controversial; and whenever I ventured to dissent from received opinions, I endeavoured to support my own positions by authorities or by facts.

It is necessarily painful to intrude upon the world personal considerations and personal feelings; it is perhaps impossible to speak much of oneself without the appearance at least, and perhaps the reality also of an egotism, which offends if it does not disgust.

I may be however permitted to remark, that I have not needlessly obtruded myself; but have rather been dragged before the public: and with that observation, which I trust will be borne in mind in estimating these remarks, I shall conclude the present communication.

I have the honor to be,

Sir,

Your most obedient Servant,

B. PHILLIPS.

*Wimpole Street.*

